# Is the Phenotype Designation by PSP-MDS Criteria Stable Throughout the Disease Course and Consistent With Tau Distribution?

**DOI:** 10.3389/fneur.2022.827338

**Published:** 2022-02-03

**Authors:** Javier Sánchez-Ruiz de Gordoa, Victoria Zelaya, Paula Tellechea-Aramburo, Blanca Acha, Miren Roldán, Carlos López-Molina, Valle Coca, Arkaitz Galbete, Maite Mendioroz, M. Elena Erro

**Affiliations:** ^1^Department of Neurology, Hospital Universitario de Navarra, Navarra Institute for Health Research (IdiSNA), Pamplona, Spain; ^2^Neuroepigenetics Laboratory-Navarrabiomed, Hospital Universitario de Navarra, Universidad Pública de Navarra (UPNA), Navarra Institute for Health Research (IdiSNA), Pamplona, Spain; ^3^Department of Pathology, Hospital Universitario de Navarra, Navarra Institute for Health Research (IdiSNA), Pamplona, Spain; ^4^Dpto. Automática y Computación, Universidad Pública de Navarra (UPNA), Navarra Institute for Health Research (IdiSNA), Pamplona, Spain; ^5^Navarrabiomed Brain Bank, Hospital Universitario de Navarra, Universidad Pública de Navarra (UPNA), Navarra Institute for Health Research (IdiSNA), Pamplona, Spain; ^6^Navarrabiomed, Universidad Pública de Navarra (UPNA), REDISSEC, Navarra Institute for Health Research (IdiSNA), Pamplona, Spain

**Keywords:** tauopathies, progressive supranuclear palsy (PSP), phenotypes, PSP-MDS criteria, tau protein load, clinical-pathological correlation

## Abstract

**Introduction:**

The MDS-PSP criteria have shown high sensitivity for the PSP diagnosis, but do not discriminate the phenotype diversity. Our purpose was to search for anatomopathological differences among PSP phenotypes resulting from the application of the MDS-PSP criteria comparing with the previous ones.

**Methods:**

Thirty-four PSP cases from a single brain bank were retrospectively classified according to the criteria used by Respondek et al. in 2014 and the PSP-MDS criteria at 3 years (MDS-3y), 6 years (MDS-6y) and at the last clinical evaluation before death (MDS-last). Semiquantitative measurement of total, cortical and subcortical tau load was compared. For comparative analysis, PSP-Richardson syndrome and PSP postural instability were grouped (PSP-RS/PI) as well as the PSP atypical cortical phenotypes (PSP-Cx).

**Results:**

Applying the Respondek's criteria, PSP phenotypes were distributed as follow: 55.9% PSP-RS/PI, 26.5% PSP-Cx, 11.8% PSP-Parkinsonism (PSP-P), and 5.9% PSP-Cerebellum. PSP-RS/PI and PSP-Cx had a higher total tau load than PSP-P; PSP-Cx showed a higher cortical tau load than PSP-RS/PI and PSP-P; and PSP-RS/PI had a higher subcortical tau load than PSP-P. Applying the MDS-3y, MDS-6y and MDS-last criteria; the PSP-RS/PI group increased (67.6, 70.6 and 70.6% respectively) whereas the PSP-Cx group decreased (8.8, and 8.8 and 11.8%). Then, only differences in total and subcortical tau burden between PSP-RS/PI and PSP-P were observed.

**Interpretation:**

After the retrospective application of the new MDS-PSP criteria, total and subcortical tau load is higher in PSP-RS/PI than in PSP-P whereas no other differences in tau load between phenotypes were found, as a consequence of the loss of phenotypic diversity.

## Introduction

Progressive supranuclear palsy (PSP) is a neurodegenerative disease characterized pathologically by the accumulation of hyperphosphorylated four-repeat (4R) tau protein within neurons (neurofibrillary tangles) and glial cells (tufted astrocytes and coiled bodies) (1).

The clinical diagnostic criteria established in 1996 by the National Institute of Neurological Disorders and Stroke (NINDS) (2) have high specificity for the diagnosis of the most frequent clinical presentation and original description of the disease, Richardson syndrome (PSP-RS), which is characterized by gait instability and falls, ophthalmoplegia, pseudobulbar signs and cognitive impairment. However, NINDS-PSP criteria have low sensitivity for the diagnosis of the atypical PSP phenotypes that have been described throughout the last 20 years, especially in early stages of the disease (3–7). For this reason, the movement disorders society (MDS) has recently reformulated PSP diagnostic criteria (8). The new MDS-PSP criteria categorize PSP symptoms into four clinical domains: ocular motor, postural instability, akinesia and cognitive dysfunction. Different combinations of these symptoms have defined various PSP phenotypes: PSP-RS, PSP-predominant postural instability (PSP-PI); PSP-predominant ocular motor dysfunction (PSP-OM); PSP-predominant parkinsonism (PSP-P); PSP-progressive gait freezing (PSP-PGF); PSP-predominant corticobasal syndrome (PSP-CBS); PSP-predominant frontal presentation (PSP-F) and PSP-predominant speech/language disorder (PSP-SL).

The pathogenic basis underlying these PSP phenotypes seems to be due to the different load and distribution of tau protein in a variety of cell types across encephalic regions (9, 10). This hypothesis is derived from studies showing more severe and widespread tau deposits in PSP-RS than in the subcortical phenotypes (3) and higher tau accumulation in neocortical regions in PSP-CBS (11), PSP-F (6), and PSP-SL (10) than in PSP-RS. In addition, to differences in overall total tau burden, clinical phenotypes can also be differentiated based on the different cell types involved (neurons, astrocytes, oligodendroglia) (10).

The application of the new MDS-PSP criteria in clinical setting has confirmed a higher sensitivity than the previous criteria (12). However, they have demonstrated a lower phenotype diversity with higher representation of PSP-RS (13) and poor accuracy in differentiating PSP-RS from PSP-P phenotypes (14), although some of these studies have not included autopsy-confirmed diagnosis (15).

Prior to the publication of the new MDS-PSP criteria, Respondek et al. (16) studied the phenotypic spectrum of PSP by retrospective chart review in a cohort of 100 autopsy-confirmed PSP patients, constituting the first quantitative description of the relative distribution of PSP clinical phenotypes defined in a large multicenter cohort.

After the MDS-PSP criteria description, the study of Kovacs et al. (10) was the first one comparing tau load among PSP phenotypes and no other similar studies have been published afterwards.

The aim of our study was to search for anatomopathological differences regarding tau load and distribution among PSP phenotypes of 34 definitive PSP cases from the same brain bank resulting from the application of the criteria applied by Respondek et al. (16) and the new MDS-PSP diagnostic criteria (8).

## Materials and Methods

### Subjects

Thirty-four pathologically confirmed PSP cases from the NavarraBiomed Brain Bank (2005–2017) were included. The study was performed under the ethics guidelines issued by our institution and written informed consent was obtained from all participants or family caregivers for pathological brain studies.

### Classification Into PSP Clinical Phenotypes

We performed a retrospective review of the clinical charts of all patients included with a clinical diagnosis of PSP. Experienced movement disorders neurologists examined all patients and the clinical items not specifically mentioned in the clinical records were considered as absent. The age of onset (age at first symptom related with PSP), disease duration (years), sex and core clinical features were recorded for each case. Patients were retrospectively allocated into the different predominant phenotypes according to the criteria applied by Respondek in 2014 (Respondek's criteria, Supplementary Table 1) (16). Moreover, they were classified into PSP-RS, PSP-PI, PSP-P, PSP-SL, PSP-F and PSP-CBS at 3 and 6 years of disease evolution (MDS-3y and MDS-6y respectively) and at the last clinical evaluation before death (MDS-last) applying the new MDS-PSP criteria (8). If a patient was deceased before that period, the data from the last evaluation was considered. The MAX rules (17) were also implemented when multiple alternatives were present for a single patient. Cases, which did not meet the clinical criteria of any phenotype, were classified as unclassified (PSP-U).

PSP-RS and PSP-PI phenotypes were grouped as PSP-RS spectrum (PSP-RS/PI) considering PSP-PI as a transitional form of PSP-RS (18). The PSP-SL, PSP-F and PSP-CBS were grouped as PSP cortical predominant phenotypes (PSP-Cx) for comparative analysis.

### Neuropathological Examination and Immunohistochemistry

Brain processing was made according to the recommendation guide proposed by BrainNet Europe (19). Routine workflow included immunohistochemical staining of 3–5 μm-thick paraffin-embedded sections, followed by counterstaining with hematoxylin-eosin. Tau pathology detection was performed with a mouse monoclonal antibody anti-human PHF-TAU, clone AT8, (Innogenetics, Ghent, Belgium). Presence of other protein deposits were analyzed using a mouse monoclonal antibody against alfa-synuclein (NCL-L-ASYN; LeicaBiosystems) or an anti-phospho TDP-43 monoclonal antibody (TIP, PTD-MO1, Cosmo Bio). Following incubation with the primary antibody, the sections were incubated with EnVision + system peroxidase (Dako, Agi- lent Technologies, Santa Clara, CA, USA) for 30 min at room temperature. The peroxidase reaction was visualized with diaminobenzidine and H_2_O_2_. Control of the immunostaining included omission of the primary antibody; no signal was obtained following incubation with only the secondary antibody. Antibody omission was used as a negative control for all staining.Neuropathological examination was completed following the revised NINDS criteria (20) for PSP diagnosis and the assessment of others co-pathologies (Alzheimer disease, Lewy bodies disease, Multiple System Atrophy, TDP 43 frontotemporal lobar degeneration and argyrophilic grain disease) were performed according to their specific recommendations (21–23).

### Regional tau Protein Density Quantification

For each subject, 21 brain regions stained with an antibody anti-human PHF-TAU, were analyzed by an investigator blinded to clinical data. These areas included: cortical regions (primary motor cortex, middle frontal cortex, parietal cortex, and their adjacent white matters), subcortical regions (putamen, globus pallidus, basal nucleus of Meynert, medial thalamus, subthalamic nucleus, substantia nigra pars compacta, midbrain tectum, ventral part of the pons, locus coeruleus and inferior olivary nucleus) and cerebellar regions (dentate nucleus, cerebellar cortex and white matter). The hippocampus region was excluded to avoid AD-related bias.

The presence of neurofibrillary tangles, neuropil threads, tufted astrocytes and oligodendroglial coiled bodies were graded using a four categories semiquantitative analysis (0 = absent, + = mild, + + = moderate, + + + = severe) (Supplementary Figure 1). Total PSP-tau burden was obtained by averaging all scores. Cortical PSP-tau and a subcortical PSP-tau burden were obtained by averaging cortical and subcortical regions respectively, excluding cerebellar structures. In addition, tau staining in neurons (tangles and neuropil threads), astrocytes and oligodendrocytes (coiled bodies and neuropil threads in the white matter) were averaged separately.

### Analysis of Genetic Variants

Genomic DNA was isolated from frozen frontal cortex tissue by phenol-chloroform method (24). *APOE* genotyping was performed using polymerase chain reaction restriction fragment length polymorphism (PCR-RFLP) (25). *APOE* genotypes were analyzed after digesting amplified sequences with 5 units of HhaI enzyme at 37°C overnight. H1/H2 haplotypes were assessed based on the *MAPT*_238 bp deletion/insertion variant located at intron 9 as previously described (26).

### Statistical Analysis

Data from continuous variables were expressed by mean ± standard deviation and categorical variables by frequencies and percentage. Cox's regression adjusted for age of onset and total tau burden were used to compare disease duration between PSP RS/PI and the other phenotypes (PSP-Cx + PSP-P). Following the recent findings of other authors (27) we also compared disease duration between PSP-P and the other phenotypes (PSP-RS/PI + PSP-Cx). Linear regressions were computed to evaluate group differences in tau burdens among PSP phenotypes adjusting by age of onset and Braak stage and to analyze the association between tau burdens and disease duration adjusting by age of onset and Braak stage. Linear regressions were also computed to evaluate the association between *MAPT* or *APOE* genotype and tau burdens or disease duration adjusting by age of onset and Braak stage. Significance level for all comparisons was set at *p* value <0.05. Statistical analysis was performed with SPSS software version 21.0 (IBM, Inc., USA).

## Results

### PSP Phenotypes Distribution

**Figure 1:**
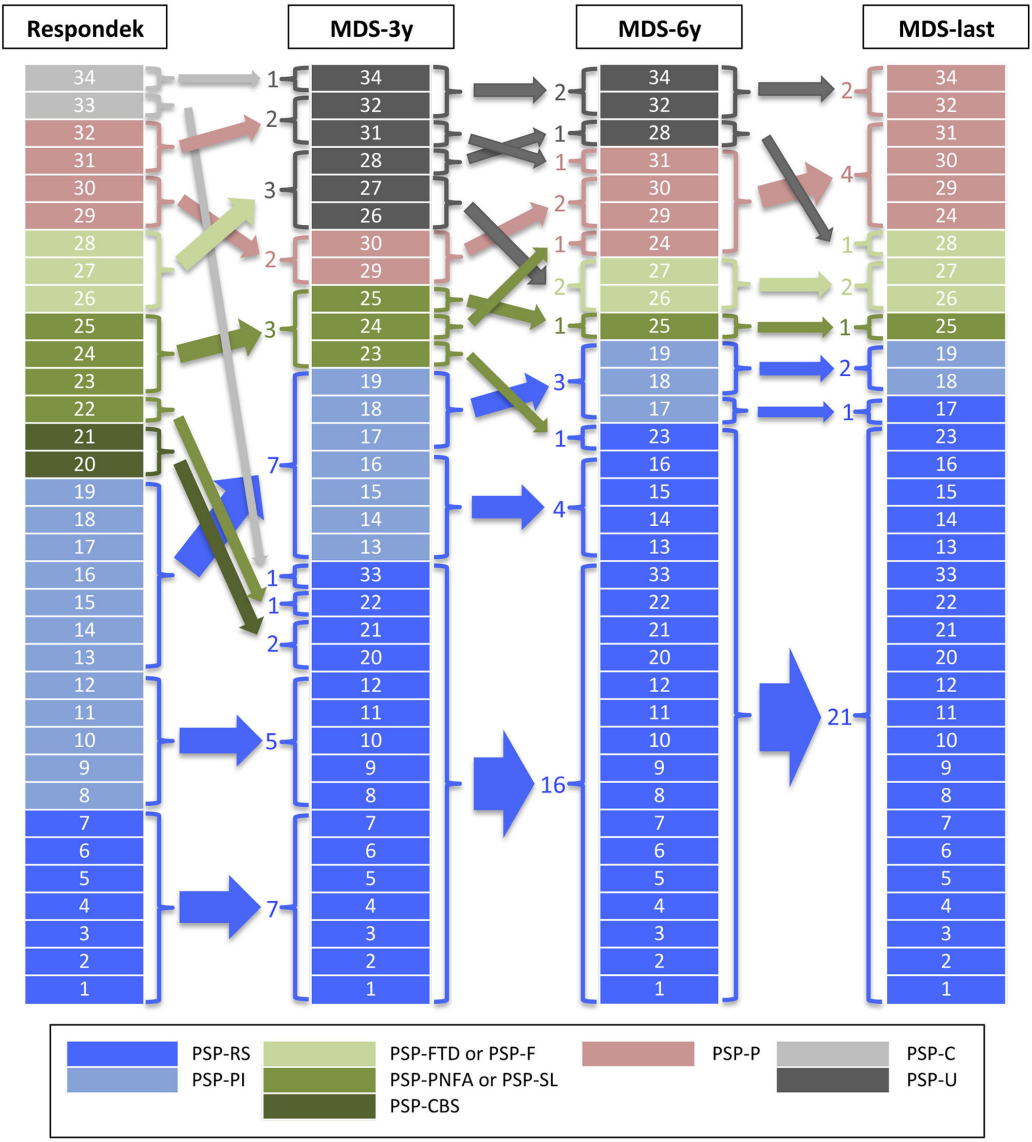
PSP phenotype distribution. The figure shows the PSP phenotype distribution after applying the criteria used by Respondek et al. in (16) (Respondek) or the Movement Disorders Society criteria and MAX rules at 3 years of disease evolution (MDS-3y), at 6 years (MDS-6y) and at last clinical evaluation before to death (MDS-last). PSP-RS, Richardson's syndrome; PSP-PI, PSP with postural instability; PSP-FTD or PSP-F, PSP with predominant frontal presentation; PSP-PNFA or PSP-SL, PSP with predominant speech/language disorder; PSP-CBS, PSP with corticobasal syndrome; PSP-P, PSP with predominant parkinsonism; PSP-C, PSP with cerebellar presentation; PSP-U, unclassified PSP.

The distribution of the PSP clinical phenotypes is summarized in Figure 1 and Supplementary Table 2. When applying the Respondek's criteria for predominant PSP phenotypes (16) we found the following distribution. 55.9% of patients exhibited a PSP-RS/PI phenotype (20.6% PSP-RS and 35.3% PSP-PI), 26.5% displayed a PSP-Cx variant (11.8% PSP-Progressive Non-Fluent Aphasia [PSP-PPNFA], 8.8% of patients had a PSP-Frontotemporal Dysfunction phenotype [PSP-FTD], and 5.9% PSP-CBS), 11.8% PSP-P, and 5.9% PSP-Cerebellum (PSP-C, not recognized in the new MDS-PSP classification). After applying the MDS-3y criteria, PSP phenotypes were distributed as follow: 67.6% PSP-RS/PI (47.1% PSP-RS and 20.6% PSP-PI), 17.6% PSP-U, 8.8% PSP-Cx (8.8% PSP-SL) and 5.9% PSP-P. Using the MDS-6y criteria, a different phenotypes distribution was obtained: 70.6% PSP-RS/PI (61.8% PSP-RS and 8.8% PSP-PI), 11.8% PSP-P, 8.8% PSP-Cx (5.9% PSP-SL and 2.9% PSP-F) and 8.8% PSP-U. Finally, applying MDS-last criteria the distribution was: 70.6% PSP-RS/PI (64.7% PSP-RS and 5.9% PSP-PI), 17.6% PSP-P, 11.8% PSP-Cx (8.8% PSP-SL and 2.9% PSP-F) and no PSP-U cases. No case ever met the criteria for PSP-PGF or PSP-OM.

### Demographics and Disease Duration

The individual patient demographic characteristics are summarized in Table 1. Briefly, 52.9% of the patients were female and the average age of onset (SD) and disease duration were 72.3 (7.3) and 8 (4) years respectively. The mean time to diagnosis (SD) was 4.6 (4.2) years and *Premortem* diagnosis accuracy rate in the last clinical evaluation was 91.2%. Case 30 and 32, classified as PSP-P according to the Respondek's and MDS-last criteria, were misdiagnosed in life as Parkinson's disease (PD); and case 25 allocated to PSP-PPNFA or PSP-SL was misdiagnosed as frontotemporal dementia.

**Table 1:** Demographic and pathological features of the sample.

**Case ID**	**Sex**	**Age of onset (yr.)**	**Disease duration (yr.)**	**Premortem diagnosis**	**Time to diagnosis (yr.)**	**Copathology**	**Braak stage**	**APOE genotype**	**MAPT genotype**
34	F	70, 8	17, 5	PSP	7, 8	AD	1	E3/E3	H1/H1
33	M	71, 9	8, 4	PSP	3, 4	AD	1	n.a.	H1/H1
32	M	68, 7	17	PD	–	AG	0	E3/E3	H1/H1
31	M	61, 8	8, 3	PSP	6	none	0	E3/E3	H1/H1
30	M	60, 3	18, 3	PD	–	AD	3	E2/E4	H1/H2
29	F	72, 4	9, 3	PSP	3, 8	AD	2	E3/E3	H1/H1
28	M	65	12	PSP	10	none	0	E3/E3	H1/H1
27	M	68, 3	11, 4	PSP	5, 3	AA	0	E3/E3	H1/H1
26	F	68, 5	9, 9	PSP	3, 5	none	0	E3/E3	H1/H1
25	F	81, 3	10, 2	FTD	–	AD	3	E3/E4	H1/H2
24	M	72, 6	9, 3	PSP	6, 5	AA	0	E3/E3	H1/H1
23	F	73, 1	6	PSP	5, 2	AD	2	E3/E4	H1/H1
22	M	73, 1	4, 1	PSP	2, 1	LBD, AG	0	E3/E3	H1/H1
21	M	64, 6	6, 2	PSP	3, 8	none	0	E3/E3	H1/H1
20	F	75, 2	10, 5	PSP	4, 4	none	0	E3/E3	H1/H2
19	F	66, 6	2, 6	PSP	2, 2	AD	2	E3/E4	H2/H2
18	M	77, 4	4, 1	PSP	1,7	LBD	0	E3/E3	H1/H1
17	F	79, 4	9, 5	PSP	7, 8	AD, AA	1	E3/E3	H1/H1
16	F	64,7	6, 9	PSP	4, 2	none	0	E3/E3	H1/H1
15	F	74	11, 6	PSP	5, 4	AD, AA	4	E3/E3	H1/H1
14	F	83, 8	4,3	PSP	2, 6	AA	0	E3/E3	H1/H1
13	M	64, 3	6	PSP	3	none	0	E3/E3	H1/H1
12	F	56, 4	7, 3	PSP	2, 6	None	0	E3/E3	H1/H1
11	M	64, 2	5, 8	PSP	3	None	0	n.a.	n.a.
10	F	78, 3	3, 8	PSP	2, 4	AD, AA	1	E3/E3	H1/H1
9	F	79, 6	11	PSP	3, 1	AD, LBD, TDP43	2	E2/E3	H1/H2
8	F	76, 6	8,6	PSP	2, 7	AD	2	E3/E3	H1/H1
7	M	77, 5	4, 5	PSP	2, 6	AA	0	E3/E3	H1/H1
6	F	88, 6	3, 4	PSP	0	AD, AA	1	n.a.	n.a.
5	M	82	4	PSP	1	None	0	E3/E3	H1/H1
4	M	73	6, 2	PSP	0, 9	AD	2	E3/E3	H1/H1
3	F	62, 1	5, 2	PSP	2, 2	None	0	E3/E3	H1/H1
2	M	79	5, 3	PSP	1, 5	AD	4	n.a.	n.a.
1	F	84	4, 4	PSP	0	AD, LBD	4	E3/E3	H1/H1

*ID, identification; yr., years; F, female; M, male; PD, Parkinson disease; FTD, frontotemporal dementia; AD, Alzheimer disease; AG, argyrophilic grains; AA, amyloid angiopathy; LBD, Lewy bodies disease; TDP43, hippocampal TDP-43 protein deposits. n.a, not available*.

The PSP-RS/PI phenotype had shorter disease duration than the other phenotypes (PSP-P + PSP-Cx) in the resultant groups after applying Respondek's and MDS-PSP criteria (MDS-6y and MDS-Last), adjusting for age at onset and total tau burden (Supplementary Table 3). When comparing PSP-P with the other phenotypes (PSP-RS + PSP-Cx), the PSP-RS/PI + PSP-Cx group had shorter disease duration than PSP-P applying MDS-last criteria with similar hazard ratio and no other significant differences were found (Supplementary Table 3).

### Neuropathological Description

The individual pathological features are summarized in Table 1. The 32.4% of the patients exhibited exclusively pathological findings of PSP. Alzheimer disease (AD) was the most frequent co-exiting pathology (47.1%), with moderate-advanced stages (Braak stage 3–6) in 14.7% of cases. α-synuclein deposits were found in 11.8% of PSP cases (4 cases), which belonged to the PSP-RS/PI phenotype independently of the moment or classifying criteria employed. One case (number 18) showed Lewy bodies limited to the brainstem, and the other 3 cases displayed a limbic-transitional distribution. In addition, in case 9, hippocampal TDP-43 protein deposits were also identified. Finally, amyloid angiopathy and limbic argyrophilic grains were found in 8 and 2 cases respectively.

### Tau Burden Distribution

**Figure 2:**
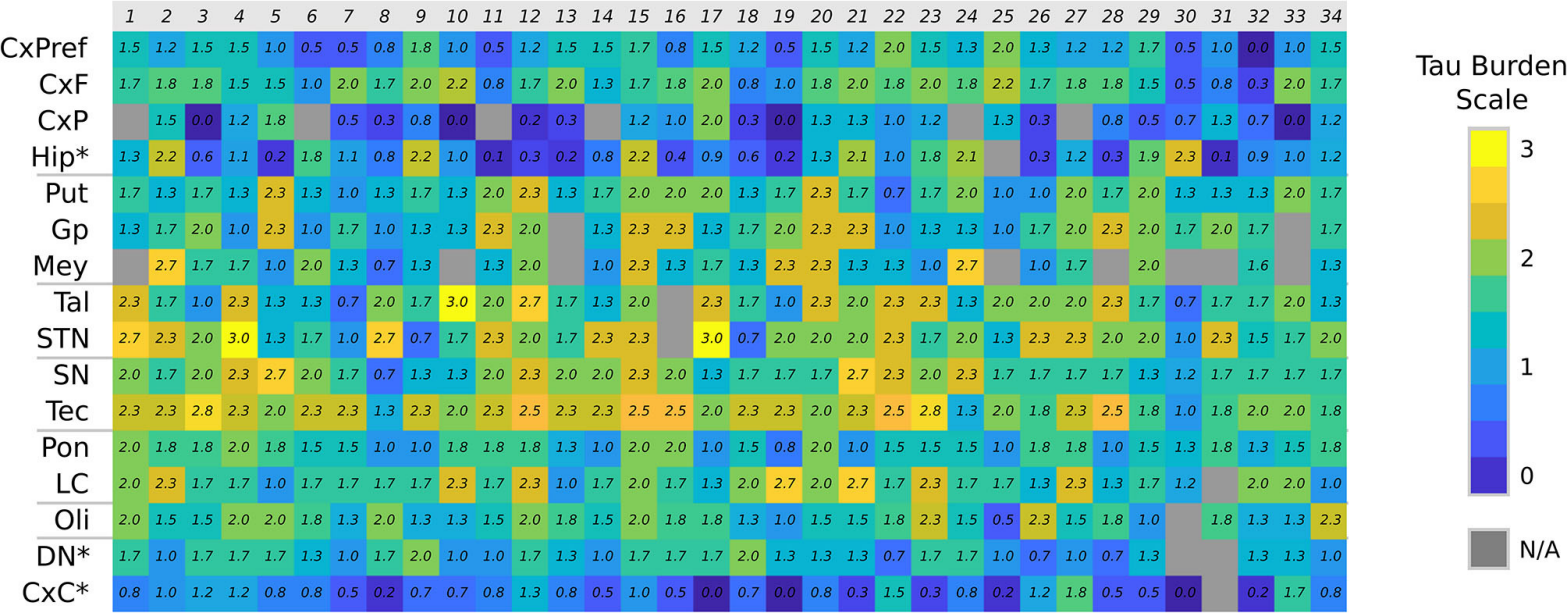
Tau burden heat map. Color chart representing the semiquantitative tau burden score of each anatomical region (CxF, primary motor cortex; CxPreF, middle frontal cortex; CxF, parietal cortex; Hip, Hippocampus; Put, putamen; Gp, globus pallidus; Mey, basal nucleus of Meynert; Tal, medial thalamus; STN, subthalamic nucleus; SN, substantia nigra; Tec, midbrain tectum; Pon, ventral part of the pons; LC, locus coeruleus; Oli, olive nucleus; DN, dentate nucleus; CxC, cerebellar cortex). N/A, not available. *Not included in the averages.

**Figure 3:**
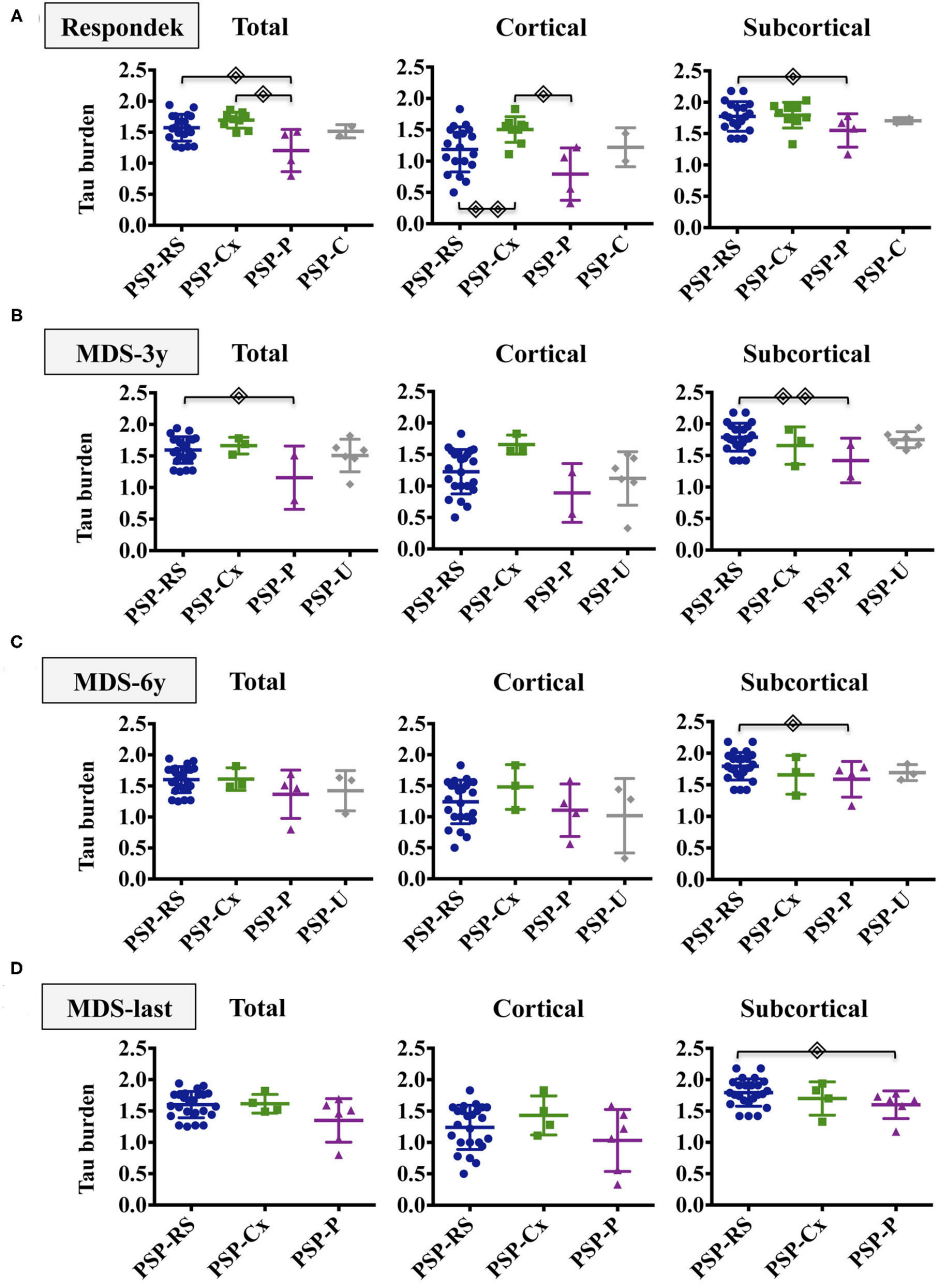
Tau burden distribution in PSP phenotypes. The figure shows the total, cortical and subcortical semiquantitative tau loads in the different PSP phenotypes after applying Respondek's **(A)**, MDS-3y **(B)**, MDS-6y **(C)** and MDS-last **(D)** criteria. Simple linear regressions were computed to evaluate group differences between PSP subtypes adjusting by age of onset and Braak stage. 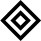 (*p* <0.05), 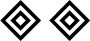 (*p* <0.01). Non-significant differences were not displayed. PSP-RS/PI, Richardson Syndrome spectrum; PSP-Cx, PSP cortical predominant phenotypes; PSP-P, PSP with predominant parkinsonism; PSP-U, unclassified PSP.

The distribution of tau load across the different brain regions of each case is represented in a heat map in Figure 2. Considering all cases, tau pathology was found more abundantly in subcortical structures. Differences in total, cortical and subcortical tau burden among phenotypes adjusted by age of onset and AD Braak stage are showed in Figures 3A–D.

Applying the Respondek's criteria (Figure 3A), PSP-RS/PI and PSP-Cx had higher total tau burden than PSP-P. Cortical tau load was significantly more abundant in PSP-Cx than in PSP-RS/PI and PSP-P whereas differences in subcortical tau burden where only found between PSP-RS/PI and PSP-P. Regarding cellular tau pathology (Supplementary Figure 2), the PSP-Cx group showed higher total and cortical neuronal tau burden than PSP-P (*p* < 0.05) and higher neuronal, astroglial and oligodendrocyte cortical tau burden than PSP-RS/PI (*p* < 0.05).

When applying the MDS-3y criteria (Figure 3B), only significant differences in total and subcortical tau burden between PSP-RS/PI and PSP-P were observed. Neuronal subcortical tau burden was more abundant in PSP-RS/PI than in PSP-P. Moreover, higher astroglial cortical tau burden was found in PSP-Cx than PSP-RS/PI (Supplementary Figure 3).

When applying MDS-6y and MDS-last criteria (Figures 3C,D), only subcortical tau load was found significantly higher in PSP-RS/PI than in PSP-P without differences in cellular tau burden.

In the simple linear regression adjusting by age of onset and AD Braak stage, there was no association between total, cortical or subcortical tau burden and disease duration in the overall sample.

### Genetic Profile of the Sample

A predominance of *MAPT* gene haplotype H1 was observed: 83.9 % of the patients presented an H1/H1 genotype, 12.9 % presented the H1/H2 genotype and only one case presented an H2/H2 genotype. Regarding the *APOE* genotype, E3/E3 was the most frequent genotype (83.3 %). Isolated cases presented haplotypes E2 (6.6 %) or E4 (13.3 %), (Table 1). There was no association between *MAPT* gene haplotype or *APOE* genotype and disease duration or tau burden.

## Discussion

The new MDS-PSP classification (8) resulted from the need to establish sensitive and specific clinical criteria for the diagnosis of the different PSP phenotypes that have been described after the 1996 definition of the NINDS-PSP criteria (2), which are very specific for the PSP-RS phenotype, but very insensitive for the atypical variants. To date, several retrospective studies validating the new MDS-PSP criteria have been published (12–15) confirming that these criteria are much more sensitive and will likely lead to better detection of PSP vs. other neurodegenerative diseases being also feasible to apply in real clinical settings. Regarding the accuracy of these sets of criteria for differentiating among the various PSP phenotypes, their usefulness has been shown to be limited since they may overestimate the presence of PSP-RS and PSP-P phenotypes in detriment of the cortical phenotypes (13). This is noteworthy, as one of the main goals of the new MDS-PSP criteria has been to allow earlier diagnosis of PSP in general, but specifically of the atypical variants of PSP. These studies, some of which have no pathological correlate, have settled the need to redefine or revise the new MDS-PSP criteria (13, 15). On the other hand, to date, there are no pathological criteria to differentiate the phenotypes of PSP, but it seems clear that they depend on the different tau load and distribution within encephalic regions and to the different patterns of cellular tau pathologies (10). Taking into account all these data, we studied the tau load and distribution among the PSP phenotypes of a series of 34 cases who were retrospectively classified following the criteria established by Respondek in 2014 and also according with phenotypes resulting from the new MDS-PSP criteria, at 3 years of disease progression, at 6 years and at the final visit before death.

Handling a small sample of patients, we have grouped all cortical phenotypes in a group (PSP-Cx) and also PSP-RS together with PSP-PI (PSP-RS-PI), since the latter has been shown to be a transitional form of PSP-RS in which it transforms in a high percentage of cases with disease progression (28).

The application of the MDS-3y criteria implies a reduction of cases fulfilling features for diagnosis of atypical phenotypes and 6 cases of PSP-U arise, mainly at the expense of cortical phenotypes, which are reduced to 3 (PSP-SL). One of the PSP-C cases also moves to the PSP-U group and the other one to the PSP-RS group. This fact may be due to a bias because of the retrospective nature of the study and the lack of clinical data on incipient involvement of oculomotor dysfunction or postural instability. The number of PSP-P cases is reduced to two, in contrast to other studies that find that MDS-3y criteria overestimates the diagnosis of this phenotype in early stages of the disease (14). The number of cases in the PSP-RS/PI group applying the PSP-MDS criteria is practically similar, regardless of whether applied at 3 years of evolution, at 6 years or at the last visit before death. Our findings are in line with other recent publications showing that the new PSP-MDS criteria tends to overestimate PSP-RS in detriment of cortical phenotypes (13). This is due to the fact that some cortical phenotypes (PSP-CBS and PSP-SL) are unable to reach a higher level of certainty than “possible” and, thus, converge to PSP-RS or PSP-P phenotypes after applying MAX rule 1 (17). Surprisingly, we found a high number of PSP-SL cases compared to other series (10, 12, 13, 16) and this fact possibly reflects a case selection bias for biobank study.

The largest differences in tau load between phenotypes are found when comparing cases resulting from the application of Respondek's criteria and thus, not only are significant differences in total and subcortical tau load found between the PSP-RS/PI and PSP-P groups, but also more cortical tau is found in the PSP-Cx group with respect to the PSP-RS/PI and PSP-P groups. The loss of phenotypic diversity implied by the application of the PSP-MDS criteria leads in our study to a loss of significant differences in tau load between the different phenotypes, contrary to the findings of the first study comparing the tau load between the phenotypes resulting from the application of the PSP-MDS criteria (10). In this multicenter study the cortical phenotypes do show increased cortical tau load. Our work does not reproduce these findings, probably because it is a single-center study with a small sample. Regarding cellular pathology after applying MDS-3y criteria, differences between PSP-Cx and PSP-RS/PI in the astroglial cortical tau load and between PSP-RS and PSP-P in the subcortical neuronal tau load were observed. Although they might be in consonance with those described by Kovacs et al. (10), they should be taken cautiously because of the phenotype clustering performed for the analysis.

Our findings, and those of other *postmortem* studies, do not allow to establish what happens in the early stages of the disease. We hypothesize that in cortical phenotypes, tau protein deposits firstly in the cortex, causing a cortical/subcortical gradient greater in the initial phases of the disease, and this fact is what determines the clinical syndrome. Unlike other neurodegenerative diseases such as AD or PD (23, 29), where different stages of disease progression have been defined, the evolution by stages in PSP is not equally stablished. Prodromal symptoms (16, 30) or preclinical forms have not been studied in depth until recently (31, 32). It has been suggested that tau pathology is initially confined to the pallid-nigro-lusian system (10, 33) and from these nuclei it extends to basal ganglia, pontine nuclei, cerebellum dentate nucleus and finally to the frontal and parietal lobes. Different patterns of involvement of subcortical circuitry, suggesting different patterns of disease spread through the brain has been proposed as the basis of the differences of PSP phenotypes (34). Further developing this hypothesis, Kovacs et al. have proposed a pattern of tau pathology with a staging system in PSP-RS that might be applicable to other PSP phenotypes (10). The spread of tau protein along neural connections is a pathogenic hypothesis not yet fully confirmed in primary tauopathies (35). Even more, this mechanism could not explain the astroglial pathology of primary tauopathies (36). PET tau imaging studies may clarify where tau deposits locate in early stages of the disease (34, 37).

Regarding disease duration, it is significantly shorter in PSP-RS/PI phenotype comparing with the other phenotypes (PSP-Cx + PSP-P), independently of the criteria applied with the exception of MDS-3y, probably due to the high presence of PSP-U cases excluded from the analyses. Recently it has been found that there is more dichotomy in terms of disease duration if PSP-RS is grouped with atypical cortical phenotypes than PSP-RS compared vs. all atypical phenotypes (27). These results have not been reproduced in our sample neither has a correlation between disease duration and tau load been found. It might be related to the lack of statistical power due to the cohort size. A significant negative correlation between tau load and disease duration (3) has suggested that more fulminant disease affects more regions, more severely from disease onset and contributes to an earlier death though probably there are other factors influencing disease duration, such as neuronal death or co-pathology (38–40).

The exact pathogenic basis that determines PSP phenotypes is not known. We have found predominance in H1/H1 and E3/E3 genotypes of *MAPT* and *APOE* genes in our series. *MAPT* gene haplotype variation has been shown to influence the risk of PSP and also to play a role in the severity of the disease (41). Cellular vulnerability (neuronal or astroglial) in PSP seems to be determined by molecular mechanisms and thus neuronal deposits are associated with brain expression of synaptic genes while astroglial deposits are associated with microglial and immune system gene expression (42).

The present study has weaknesses. One limitation is its retrospective nature. The main limitation is the small sample, which implies that atypical phenotypes are underrepresented, and leads to the phenotype or the anatomical regions clustering. In addition, the percentage of cases with multiproteinopathy is high, but this fact, is in accordance with recent studies that confirm the less important impact of co-pathologies on PSP progression (39) rate and demonstrates that tau burden is the strongest correlate with clinical manifestations (40). In contrast, this study analyzes cases from the same brain biobank, which confers the strength of the standardization of neuropathological procedures.

As a conclusion, our study shows that tau load remains different between the PSP-RS and the PSP-P phenotypes resulting from retrospective application of the new MDS-PSP criteria, whereas no other differences regarding cortical or subcortical tau load between phenotypes are found, as a consequence of the loss of phenotypic diversity. Further studies are needed to understand the pathogenic basis of the different PSP phenotypes and also to confirm the need of reformulating the new MDS-PSP criteria.

## Data Availability Statement

The raw data supporting the conclusions of this article will be made available by the authors, without undue reservation.

## Ethics Statement

The studies involving human participants were reviewed and approved by Comité Ético de Investigación con medicamentos (CEIm) de Navarra. The patients/participants provided their written informed consent to participate in this study.

## Author Contributions

JS-R, ME, and MM contributed to conception and design of the study. VC, MR, JS-R, and VZ developed brain processing, sample genotyping, and measurement of tau burdens. JS-R, CL-M, and AG organized the database, designed the figures, and performed the statistical analysis. JS-R and ME wrote the first draft of the manuscript. VZ, PT-A, and BA wrote sections of the manuscript. All authors contributed to manuscript revision, read, and approved the submitted version.

## Conflict of Interest

BA is supported by a PFIS fellowship from the Spanish Government (FI18/00150) and MM received a grant “Programa de intensificación” founded by “LaCaixa Foundation” and Fundación Caja-Navarra. JS reports support for training from Pfizer. ME reports support for training from Abbvie. The remaining authors declare that the research was conducted in the absence of any commercial or financial relationships that could be construed as a potential conflict of interest.

## Publisher's Note

All claims expressed in this article are solely those of the authors and do not necessarily represent those of their affiliated organizations, or those of the publisher, the editors and the reviewers. Any product that may be evaluated in this article, or claim that may be made by its manufacturer, is not guaranteed or endorsed by the publisher.

## References

[1] Steele JC Richardson JC Olszewski J. Progressive supranuclear palsy. a heterogeneous degeneration involving the brain stem, basal ganglia and cerebellum with vertical gaze and pseudobulbar palsy, nuchal dystonia and dementia. Arch Neurol. (1964) 10:333–59. 10.1001/archneur.1964.00460160003001 14107684

[2] Litvan I Agid Y Calne D Campbell G Dubois B Duvoisin RC. Clinical research criteria for the diagnosis of progressive supranuclear palsy (steele-richardson-olszewski syndrome): report of the NINDS-SPSP international workshop^*^. Neurology. (1996) 47:1–9. 10.1212/WNL.47.1.1 8710059

[3] Williams DR de Silva R Paviour DC Pittman A Watt HC Kilford L. Characteristics of two distinct clinical phenotypes in pathologically proven progressive supranuclear palsy: Richardson's Syndrome and PSP-Parkinsonism. Brain. (2005) 128:1247–58. 10.1093/brain/awh488 15788542

[4] Williams DR Holton JL Strand K Revesz T Lees AJ. Pure akinesia with gait freezing: a third clinical phenotype of progressive supranuclear palsy. Mov Disord. (2007) 22:2235–41. 10.1002/mds.21698 17712855

[5] Ling H Ling H de Silva R Massey LA Courtney R Hondhamuni G. Characteristics of progressive supranuclear palsy presenting with corticobasal syndrome: a cortical variant. Neuropathol Appl Neurobiol. (2014) 40:149–63. 10.1111/nan.12037 23432126 PMC4260147

[6] Sakae N Josephs KA Litvan I Murray ME Duara R Uitti RJ. Neuropathologic basis of frontotemporal dementia in progressive supranuclear palsy. Mov Disord. (2019) 34:1655–62. 10.1002/mds.27816 31433871 PMC6899964

[7] Mochizuki A Ueda Y Komatsuzaki Y Tsuchiya K Arai T Shoji S. Progressive supranuclear palsy presenting with primary progressive aphasia–clinicopathological report of an autopsy case. Acta Neuropathol. (2003) 105:610–4. 10.1007/s00401-003-0682-5 12669238

[8] Höglinger GU Respondek G Stamelou M Kurz C Josephs KA Lang AE. Clinical diagnosis of progressive supranuclear palsy: the movement disorder society criteria. Mov Disord. (2017) 32:853–64. 10.1002/mds.26987 28467028 PMC5516529

[9] Dickson DW Ahmed Z Algom AA Tsuboi Y Josephs KA. Neuropathology of variants of progressive supranuclear palsy. Curr Opin Neurol. (2010) 23:394–400. 10.1097/WCO.0b013e32833be924 20610990

[10] Kovacs GG Lukic MJ Irwin DJ Arzberger T Respondek G Lee EB. Distribution patterns of tau pathology in progressive supranuclear palsy. Acta Neuropathol. (2020) 20:2. 10.1007/s00401-020-02158-2 32383020 PMC7360645

[11] Tsuboi Y Josephs KA Boeve BF Litvan I Caselli RJ Caviness JN. Increased tau burden in the cortices of progressive supranuclear palsy presenting with corticobasal syndrome. Mov Disord. (2005) 20:982–8. 10.1002/mds.20478 15834857

[12] Ali F Martin PR Botha H Ahlskog JE Bower JH Masumoto JY. Sensitivity and specificity of diagnostic criteria for progressive supranuclear palsy. Mov Disord. (2019) 34:1144–53. 10.1002/mds.27619 30726566 PMC6688972

[13] Frank A Peikert K Linn J Brandt MD Hermann A. MDS criteria for the diagnosis of progressive supranuclear palsy overemphasize richardson syndrome. Ann Clin Transl Neurol. (2020) 20:51065. 10.1002/acn3.51065 32735745 PMC7480918

[14] Shoeibi A Litvan I Juncos JL Bordelon Y Riley D Standaert D. Are the international parkinson disease and movement disorder society progressive supranuclear palsy (IPMDS-PSP) diagnostic criteria accurate enough to differentiate common PSP phenotypes? Parkinsonism Relat Disord. (2019) 69:34–9. 10.1016/j.parkreldis.2019.10.012 31665686 PMC6914266

[15] Picillo M Erro R Cuoco S Tepedino MF Manara R Pellecchia MT. MDS PSP criteria in real-life clinical setting: motor and cognitive characterization of subtypes. Mov Disord. (2018) 33:1361–5. 10.1002/mds.27408 29984518

[16] Respondek G Stamelou M Kurz C Ferguson LW Rajput A Chiu WZ. The phenotypic spectrum of progressive supranuclear palsy: a retrospective multicenter study of 100 definite cases. Mov Disord. (2014) 29:1758–66. 10.1002/mds.26054 25370486

[17] Grimm M-J Respondek G Stamelou M Arzberger T Ferguson L Gelpi E. How to apply the movement disorder society criteria for diagnosis of progressive supranuclear palsy. Mov Disord. (2019) 34:1228–32. 10.1002/mds.27666 30884545 PMC6699888

[18] Kurz C Ebersbach G Respondek G Giese A Arzberger T Höglinger GU. An autopsy-confirmed case of progressive supranuclear palsy with predominant postural instability. Acta Neuropathol Commun. (2016) 4:120. 10.1186/s40478-016-0391-7 27842578 PMC5109838

[19] Bell JE Alafuzoff I Al-Sarraj S Arzberger T Bogdanovic N Budka H. Management of a twenty-first century brain bank: experience in the brainnet Europe consortium. Acta Neuropathol. (2008) 115:497–507. 10.1007/s00401-008-0360-8 18365220

[20] Litvan I Hauw JJ Bartko JJ Lantos PL Daniel SE Horoupian DS. Validity and reliability of the preliminary NINDS neuropathologic criteria for progressive supranuclear palsy and related disorders. J Neuropathol Exp Neurol. (1996) 55:97–105. 10.1097/00005072-199601000-00010 8558176

[21] Saito Y Ruberu NN Sawabe M Arai T Tanaka N Kakuta Y. Staging of argyrophilic grains: an age-associated tauopathy. J Neuropathol Exp Neurol. (2004) 63:911–8. 10.1093/jnen/63.9.911 15453090

[22] Mackenzie IRA Neumann M Bigio EH Cairns NJ Alafuzoff I Kril J. Nomenclature and nosology for neuropathologic subtypes of frontotemporal lobar degeneration: an update. Acta Neuropathol. (2010) 119:1–4. 10.1007/s00401-009-0612-2 19924424 PMC2799633

[23] Montine TJ Phelps CH Beach TG Bigio EH Cairns NJ Dickson DW. National institute on aging-alzheimer's association guidelines for the neuropathologic assessment of Alzheimer's disease: a practical approach. Acta Neuropathol. (2012) 123:1–11. 10.1007/s00401-011-0910-3 22101365 PMC3268003

[24] Miller SA Dykes DD Polesky HF. A simple salting out procedure for extracting DNA from human nucleated cells. Nucleic Acids Res. (1988) 16:1215. 10.1093/nar/16.3.1215 3344216 PMC334765

[25] Hixson JE Vernier DT. Restriction isotyping of human apolipoprotein E by gene amplification and cleavage with HhaI. J Lipid Res. (1990) 31:545–548. 10.1016/S0022-2275(20)43176-1 2341813

[26] Baker M Litvan I Houlden H Adamson J Dickson D Perez-Tur J. Association of an extended haplotype in the tau gene with progressive supranuclear palsy. Hum Mol Genet. (1999) 8:711–5. 10.1093/hmg/8.4.711 10072441

[27] Guasp M Molina-Porcel L Painous C Caballol N Camara A Perez-Soriano A. Association of PSP phenotypes with survival: a brain-bank study. Parkinsonism Relat Disord. (2021) 84:77–81. 10.1016/j.parkreldis.2021.01.015 33581485

[28] Grimm M-J Respondek G Stamelou M Arzberger T Ferguson L Gelpi E. Clinical conditions “suggestive of progressive supranuclear palsy”—diagnostic performance. Movement Disorders. (2020) 35:230113. 10.1002/mds.28263 32914550 PMC7953080

[29] Braak H Del Tredici K Rüb U de Vos RAI Jansen Steur ENH Braak E. Staging of brain pathology related to sporadic Parkinson's disease. Neurobiol Aging. (2003) 24:197–211. 10.1016/S0197-4580(02)00065-9 12498954

[30] Nogami A Yamazaki M Saito Y Hatsuta H Sakiyama Y Takao M. Early stage of progressive supranuclear palsy: a neuropathological study of 324 consecutive autopsy cases. J Nippon Med Sch. (2015) 82:266–73. 10.1272/jnms.82.266 26823029

[31] Evidente VGH Adler CH Sabbagh MN Connor DJ Hentz JG Caviness JN. Neuropathological findings of PSP in the elderly without clinical PSP: possible incidental PSP? Parkinsonism Relat Disord. (2011) 17:365–71. 10.1016/j.parkreldis.2011.02.017 21420891 PMC3109165

[32] Painous C Martí MJ Simonet C Garrido A Valldeoriola F Muñoz E. Prediagnostic motor and non-motor symptoms in progressive supranuclear palsy: the step-back PSP study. Parkinsonism Relat Disord. (2020) 74:67–73. 10.1016/j.parkreldis.2020.03.003 32536421

[33] Yoshida K Hata Y Kinoshita K Takashima S Tanaka K Nishida N. Incipient progressive supranuclear palsy is more common than expected and may comprise clinicopathological subtypes: a forensic autopsy series. Acta Neuropathol. (2017) 133:809–23. 10.1007/s00401-016-1665-7 28064358

[34] Whitwell JL Tosakulwong N Botha H Ali F Clark HM Duffy JR. Brain volume and flortaucipir analysis of progressive supranuclear palsy clinical variants. Neuroimage Clin. (2020) 25:102152. 10.1016/j.nicl.2019.102152 31935638 PMC6961761

[35] Mudher A Colin M Dujardin S Medina M Dewachter I Alavi Naini SM. What is the evidence that tau pathology spreads through prion-like propagation? Acta Neuropathologica Communications. (2017) 5:99. 10.1186/s40478-017-0488-7 29258615 PMC5735872

[36] Colin M Dujardin S Schraen-Maschke S Meno-Tetang G Duyckaerts C Courade J-P. From the prion-like propagation hypothesis to therapeutic strategies of anti-tau immunotherapy. Acta Neuropathol. (2020) 139:3–25. 10.1007/s00401-019-02087-9 31686182 PMC6942016

[37] Whitwell JL Höglinger GU Antonini A Bordelon Y Boxer AL Colosimo C. Radiological biomarkers for diagnosis in PSP: where are we and where do we need to be? Mov Disord. (2017) 32:955–71. 10.1002/mds.27038 28500751 PMC5511762

[38] Schofield EC Hodges JR Bak TH Xuereb JH Halliday GM. The relationship between clinical and pathological variables in richardson's syndrome. J Neurol. (2012) 259:482–90. 10.1007/s00415-011-6205-8 21837549

[39] Jecmenica Lukic M Kurz C Respondek G Grau-Rivera O Compta Y Gelpi E. Copathology in progressive supranuclear palsy: does it matter? Mov Disord. (2020) 20:28011. 10.1002/mds.28011 32125724

[40] Robinson JL Yan N Caswell C Xie SX Suh E Van Deerlin VM. Primary tau pathology, not copathology, correlates with clinical symptoms in PSP and CBD. J Neuropathol Exp Neurol. (2020) 79:296–304. 10.1093/jnen/nlz141 31999351 PMC7036659

[41] Heckman MG Brennan RR Labbé C Soto AI Koga S DeTure MA. Association of MAPT subhaplotypes with risk of progressive supranuclear palsy and severity of tau pathology. JAMA Neurol. (2019) 76:710–7. 10.1001/jamaneurol.2019.0250 30882841 PMC6563568

[42] Allen M Wang X Serie DJ Strickland SL Burgess JD Koga S. Divergent brain gene expression patterns associate with distinct cell-specific tau neuropathology traits in progressive supranuclear palsy. Acta Neuropathol. (2018) 136:709–27. 10.1007/s00401-018-1900-5 30136084 PMC6208732

