# Supplementary material for: Is the Phenotype Designation by PSP-MDS Criteria Stable Throughout the Disease Course and Consistent With Tau Distribution?
Source: Front Neurol. 2022 Feb 3;13:827338. doi: 10.3389/fneur.2022.827338 (PMC8850262; doi:10.3389/fneur.2022.827338)
Supplement: Supplementary file 1 [file Data_Sheet_1.PDF]

SUPPLEMENTARY MATERIAL

Supplementary figure 1.

Tau burden semi-quantitative analysis in the different cellular structures.

Images obtained at 10X magnification with immunohistochemistry for hyperphosphorylated tau

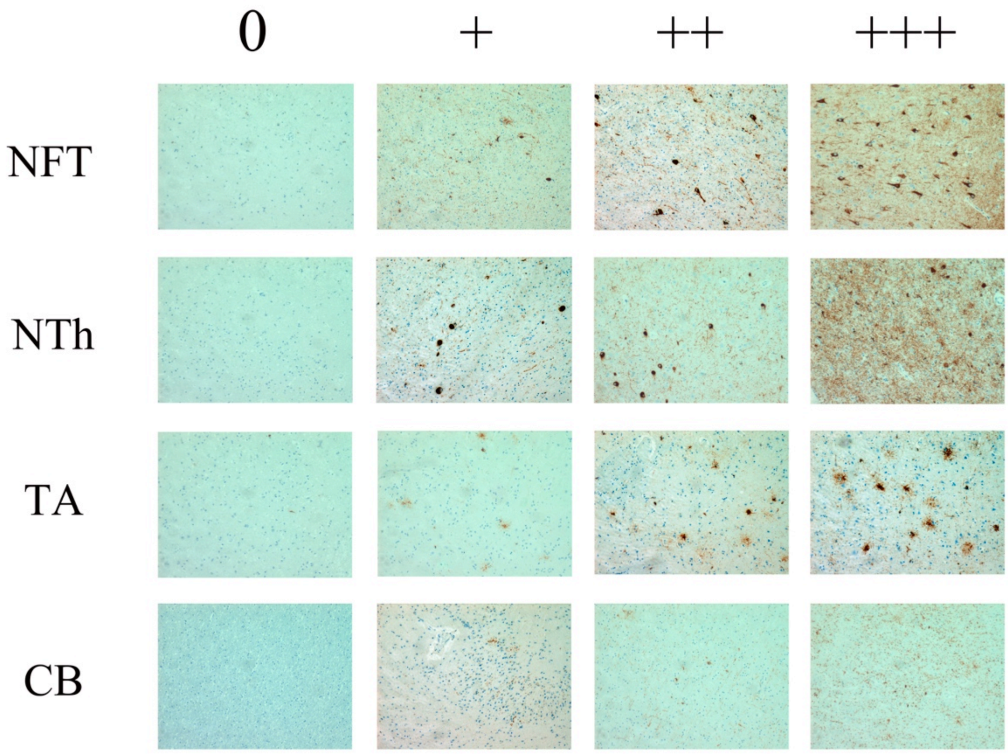

(AT8). Neurofibrillar tangles (NFT), Neuropyl threads (NTh), Tuffted Astrocytes (TA) and Coiled bodies (CB) were measured. Intensity of deposits: 0 (absent), +(mild), +++ (moderate), +++(severe). Scale bar: 500 µm**.**

Supplementary figure 2.

RESPONDEK

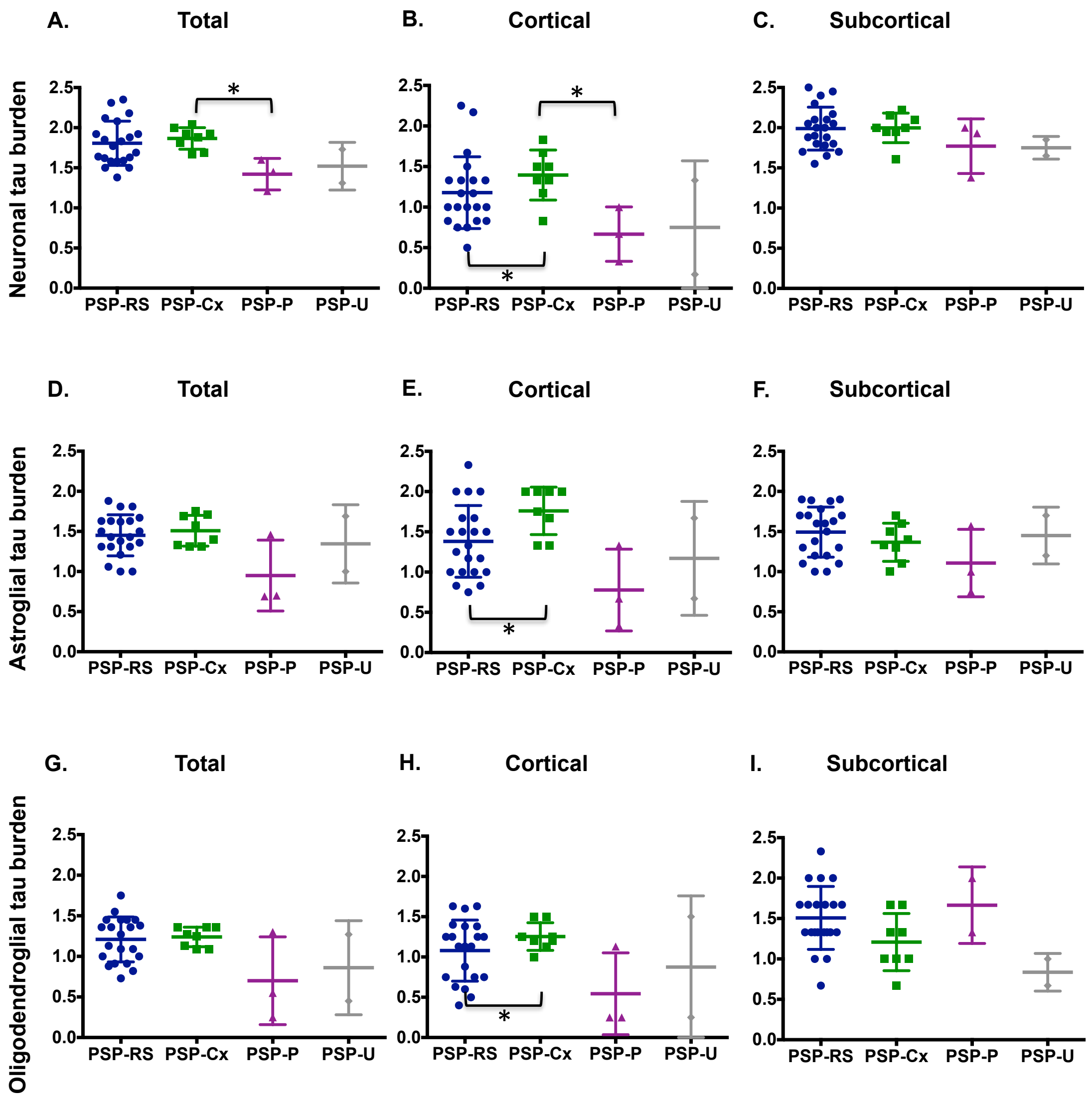

Supplementary figure 3.

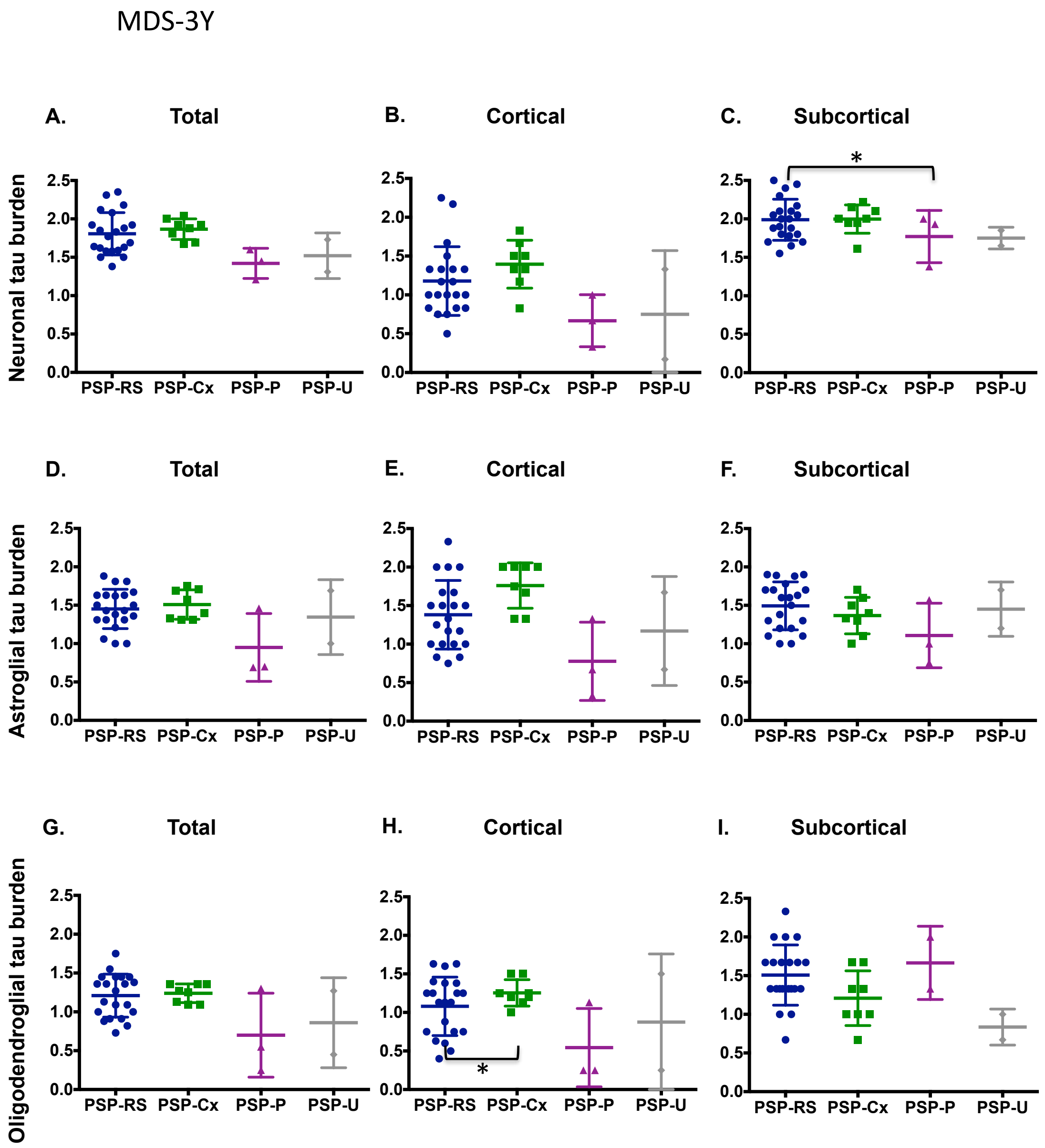

Supplementary Table 1. Respondek's criteria of predominant phenotypes of PSP.

Group	Feature	Impact
**All subtypes**	***All of the following:***	Mandatory
	Adult onset	
	Progression	
PSP-RS	***All of the following within the first 2 years:***	Predominant
	Postural instability	
	Falls	
	Abnormal saccades	
	Supranuclear gaze palsy	
PSP-PI	***All of the following within the first 2 years:***	Predominant
	Postural instability with falls	
	**No** supranuclear gaze palsy or abnormal saccades	
PSP-OM	***All of the following within the first 2 years:***	Predominant
	Supranuclear gaze palsy or abnormal saccades	
	**No** postural instability with falls	
PSP-P	***At least two of the following:***	Predominant
	Bradykinesia (any time)	
	Tremor (within the first 2 years)	
	Limb Rigidity (within the first 2 years)	
	Asymmetry at onset	Supportive
	Levodopa responsiveness	
	Absence of all of the following within the first 2 years:	Supportive
	Falls	
	Postural instability	
	Supranuclear gaze palsy<br>Abnormal saccades	
	Frontal lobe dysfunction	
PSP-PAGF	***All of the following within the first 2 years:***	Predominant
	Bradykinesia	
	Freezing of gait or speech	
	Absence of all of the following within the first 2 years:	Supportive
	Tremor<br>Rigidity	
	Supranuclear gaze palsy and abnormal saccades	

	Falls and postural instability	
PSP-CBS	***At least one extrapyramidal symptom:***	Predominant
	Bradykinesia	
	Rigidity limbs > axial	
	Dystonia axial or extra-axial	
	At least one cortical symptom:	
	Apraxia of limb(s)	
	Myoclonus	
	Cortical sensory loss	
	Alien limb phenomenon	
	Asymmetry at onset	Supportive
	Persistent Asymmetry	
	No levodopa responsiveness	
PSP-FTD	***Presence within the first 2 years of at least two of the following categories:***	Predominant
	Frontal-type personality change	
	Social dysfunction	
	Executive dysfunction	
	Frontal behavior	
	Frontal physical signs	
	Presence of all of the following within the first 2 years:	Supportive
	Cognitive dysfunction	
	No AD-like deficits	
	No postural instability or falls	
	No supranuclear gaze palsy or abnormal saccades	
PSP-PNFA	***At least one of the following:***	Predominant
	Progressive non-fluent aphasia	
	Apraxia of speech	
	Absence of all of the following within the first 2 years:	Supportive
	Falls or postural instability	
	Supranuclear gaze palsy or abnormal saccades	
PSP-SD	***Presence in the first 2 years:***	Predominant
	Semantic dementia	
PSP-C	***Presence in the first 2 years:***	Predominant
	Cerebellar ataxia	

(Adapted from Respondek et al., 2014)

RS, Richardson's syndrome; PI, Postural Instability; OM, Oculomotor; P, Parkinsonism; PAGF, Pure Akinesia with Gait Freezing; CBS, Corticobasal Syndrome; FTD, Frontotemporal Dysfunction; PNFA, Progressive Non-Fluent Aphasia; SD, Semantic Dementia; C, Cerebellar.

Supplementary Table 2. Criteria application.

Case ID	Respondek	MDS-3y			MDS-6y			MDS-last		
		MDS criteria (O, P, A, C)	Predominance subtype and certain grade	MAX rules	MDS criteria (O, P, A, C)	Predominance subtype and certain grade	MAX rules	MDS criteria (O, P, A, C)	Predominance subtype and certain grade	MAX rules
34	PSP-C	O0, P2, A0, C0	unclassified	-	O0, P2, A0, C0	unclassified	-	O1, P2, A2, C3, CC2, CC3	**prob. PSP-RS**, poss. PSP-CBS, prob. PSP-P	MAX 1, 2
33	PSP-C	O2, O3, P1, A0, C0, CC2, CC3	prob. PSP-RS	-	O1, O3, P1, A2, C0, CC2, CC3	prob. PSP-RS	-	O1, O3, P1, A2, C0, CC2, CC3	**prob. PSP-RS**, prob. PSP-P	MAX 2
32	PSP-P	O0, P0, A3, C0	unclassified	-	O0, P0, A3, C0	unclassified	-	O3, P0, A2, A3, C0, CC2, CC3	s.o. PSP-P	-
31	PSP-P	O0, P0, A3, C0	unclassified	-	O1, P0, A2, A3, C0, CC2	prob. PSP-P	-	O1, P0, A2, A3, C0, CC2, CC3	prob. PSP-P	-
30	PSP-P	O0, P2, A3, C0	s.o. PSP-P	-	O0, P2, A3, C0	s.o. PSP-P	-	O0, P0, A2, A3, C0, CC2, CC3	s.o. PSP-P	-
29	PSP-P	O0, P0, A2, C0 CC1	s.o. PSP-P	-	O0, P0, A2, C0 CC1	s.o. PSP-P	-	O3, P0, A2, C0, CC2	s.o. PSP-P	-
28	PSP-FTD	O0, P0, A0, C2	unclassified	-	O0, P0, A0, C2	unclassified	-	O1, P0, A0, C2, CC2, CC3	prob. PSP-F	-
27	PSP-FTD	O0, P0, A0, C2	unclassified	-	O1, P0, A2, C2, CC2, CC3	**prob. PSP-F**, prob. PSP-P	MAX 2	O1, P0, A2, C2, CC2, CC3	**prob. PSP-F**, prob. PSP-P	MAX 2
26	PSP-FTD	O0, P0, A0, C2	unclassified	-	O1, P0, A2, C2, CC2, CC3	**prob. PSP-F**, prob. PSP-P	MAX 2	O1, P0, A2, C2, CC2, CC3	**prob. PSP-F**, prob. PSP-P	MAX 2
25	PSP-PPNFA	O0, P,0, A0, C1	s.o. PSP-SL	-	O0, P,0, A0, C1	s.o. PSP-SL	-	O0, P0, A2, C1, CC2, CC3	**s.o. PSP-SL**, s.o. PSP-P	MAX 2
24	PSP-PPNFA	O0, P0, A0, C1, CC2	s.o. PSP-SL	-	O1, P0, A2, C1, CC2	poss. PSP-SL, **prob. PSP-P**	MAX 1	O1, P0, A2, C1, CC2, CC3	poss. PSP-SL, **prob. PSP-P**	MAX 1
23	PSP-PPNFA	O0, P1, A2, C1, CC2	**s.o. PSP-SL**, s.o. PSP-P, s.o. PSP-PI	MAX 2	O1, P1, A2, C1, CC2, CC3	poss. PSP-SL, **prob. PSP-RS**, prob. PSP-P	MAX 1, 3	O1, P1, A2, C1, CC2, CC3	poss. PSP-SL, **prob. PSP-RS**, prob. PSP-P	MAX 1, 3
22	PSP-PPNFA	O1, P1, A0, C1	**prob. PSP-RS**, poss. PSP-SL	MAX 1	O1, P1, A2, C1, CC2, CC3	poss. PSP-SL, **prob. PSP-RS**, prob. PSP-P	MAX 1, 3	O1, P1, A2, C1, CC2, CC3	poss. PSP-SL, **prob. PSP-RS**, prob. PSP-P	MAX 1, 3
21	PSP-CBS	O1, P1, A0, C3	poss. PSP-CBS, **prob. PSP-RS**	MAX 1	O1, O3, P1, A2, C3, CC2, CC3	poss. PSP-CBS, **prob. PSP-RS**, prob. PSP-P	MAX 1, 3	O1, O3, P1, A2, C3, CC2, CC3	poss. PSP-CBS, **prob. PSP-RS**, prob. PSP-P	MAX 1, 3
20	PSP CBS	O2, P1, A0, C3, CC2	**prob. PSP-RS**, poss. PSP-CBS	MAX 1	O2, P1, A2, C3, CC2	poss. PSP-CBS, **prob. PSP-RS**, prob. PSP-P	MAX 1, 3	O1, O3, P1, A2, C3, CC2, CC3	poss. PSP-CBS, **prob. PSP-RS**, prob. PSP-P	MAX 1, 3
19	PSP-PI	O0, P2, A2, C0	**s.o. PSP-PI**, s.o. PSP-P	MAX 3	O0, P2, A2, C0, CC2	**s.o. PSP-PI**, s.o. PSP-P	MAX 2	O0, P2, A2, C0, CC2	**s.o. PSP-PI**, s.o. PSP-P	MAX 2
18	PSP-PI	O0, P1, A2, C0, CC1	**s.o. PSP-PI**, s.o. PSP-P	MAX 3	O0, P1, A2, C2, CC1, CC3	**s.o. PSP-PI**, s.o. PSP-P, s.o. PSP-F	MAX 3	O0, P1, A2, C2, CC1, CC3	**s.o. PSP-PI**, s.o. PSP-P, s.o. PSP-F	MAX 3
17	PSP-PI	O0, P1, A0, C0	s.o. PSP-PI	-	O0, P1, A2, C0	**s.o. PSP-P**I, s.o. PSP-P	MAX 2	O1, O3, P1, A2, C0, CC2, CC3	**prob. PSP-RS**, prob. PSP-P	MAX 3
16	PSP-PI	O0, P1, A2, C0	**s.o. PSP-P**I, s.o. PSP-P	MAX 3	O1, P1, A2, C0, CC2, CC3	**prob. PSP-RS**, prob. PSP-P	MAX 3	O1, P1, A2, C0, CC2, CC3	**prob. PSP-RS**, prob. PSP-P	MAX 3
15	PSP-PI	O0, P1, A2, C0	**s.o. PSP-P**I, s.o. PSP-P	MAX 3	O1, P1, A2, C0, CC3	**prob. PSP-RS**, prob. PSP-P	MAX 3	O1, P1, A2, C0, CC2, CC3	**prob. PSP-RS**, prob. PSP-P	MAX 3
14	PSP-PI	O0, P1, A0, C0	s.o. PSP-PI	-	O1, O3, P1, A2, C0, CC2, CC3	**prob. PSP-RS**, prob. PSP-P	MAX 3	O1, O3, P1, A2, C0, CC2, CC3	**prob. PSP-RS**, prob. PSP-P	MAX 3
13	PSP-PI	O0, P1, A0, C0	s.o. PSP-PI	-	O1, P1, A2, C0, CC2, CC3	**prob. PSP-RS**, prob. PSP-P	MAX 3	O1, P1, A2, C0, CC2, CC3	**prob. PSP-RS**, prob. PSP-P	MAX 3
12	PSP-PI	O1, P1, A0, C2	**prob. PSP-RS**, prob. PSP-F	MAX 2	O1, P1, A2, C2, CC2, CC3	**prob. PSP-RS**, prob. PSP-F, prob. PSP-P	MAX 2	O1, P1, A2, C2, CC2, CC3	**prob. PSP-RS**, prob. PSP-F, prob. PSP-P	MAX 2
11	PSP-PI	O1, P1, A0, C0	prob. PSP-RS	-	O1, P1, A0, C0, CC2, CC3	prob. PSP-RS	-	O1, P1, A0, C0, CC2, CC3	prob. PSP-RS	-
10	PSP-PI	O1, P1, A2, C0	**prob. PSP-RS**, prob. PSP-P	MAX 3	O1, P1, A2, C0, CC2, CC3	**prob. PSP-RS**, prob. PSP-P	MAX 3	O1, P1, A2, C0, CC2, CC3	**prob. PSP-RS**, prob. PSP-P	MAX 3
9	PSP-PI	O1, P1, A2, C0	**prob. PSP-RS**, prob. PSP-P	MAX 3	O1, P1, A2, C0	**prob. PSP-RS**, prob. PSP-P	MAX 3	O1, P1, A2, C0, CC3	**prob. PSP-RS**, prob. PSP-P	MAX 3
8	PSP-PI	O1, P1, A0, C0	prob. PSP-RS	-	O1, O3, P1, A2, C0, CC2	**prob. PSP-RS**, prob. PSP-P	MAX 2	O1, O3, P1, A2, C0, CC2, CC3	**prob. PSP-RS**, prob. PSP-P	MAX 2
7	PSP-RS	O1, P1, A3, C0	**prob. PSP-RS**, prob. PSP-P	MAX 3	O1, P1, A2, A3 C0	**prob. PSP-RS**, prob. PSP-P	MAX 3	O1, P1, A2, A3 C0	**prob. PSP-RS**, prob. PSP-P	MAX 3
6	PSP-RS	O1, P1, A0, C0	prob. PSP-RS	-	O1, P1, A2, C0	**prob. PSP-RS**, prob. PSP-P	MAX 3	O1, P1, A2, C0	**prob. PSP-RS**, prob. PSP-P	MAX 3
5	PSP-RS	O1, P1, A2, C0, CC2	**prob. PSP-RS**, prob. PSP-P	MAX 3	O1, P1, A2, C0, CC2	**prob. PSP-RS**, prob. PSP-P	MAX 3	O1, P1, A2, C0, CC2	**prob. PSP-RS**, prob. PSP-P	MAX 3
4	PSP-RS	O1, P1, A2, C0, CC2, CC3	**prob. PSP-RS**, prob. PSP-P	MAX 3	O1, P1, A2, C0, CC2, CC3	**prob. PSP-RS**, prob. PSP-P	MAX 3	O1, O3, P1, A2, C0, CC2, CC3	**prob. PSP-RS**, prob. PSP-P	MAX 3
3	PSP-RS	O1, P1, A0, C0, CC2, CC3	prob. PSP-RS	-	O1, O3, P1, A2, C0, CC2, CC3	**prob. PSP-RS**, prob. PSP-P	MAX 2	O1, O3, P1, A2, C0, CC2, CC3	**prob. PSP-RS**, prob. PSP-P	MAX 2
2	PSP-RS	O1, P1, A0, C0	prob. PSP-RS	-	O1, P1, A2, C0, CC2, CC3	**prob. PSP-RS**, prob. PSP-P	MAX 2	O1, P1, A2, C0, CC2, CC3	**prob. PSP-RS**, prob. PSP-P	MAX 2
1	PSP-RS	O1, P1, A2, C0	**prob. PSP-RS**, prob. PSP-P	MAX 3	O1, O3, P1, A2, C0, CC2, CC3	**prob. PSP-RS**, prob. PSP-P	MAX 2	O1, O3, P1, A2, C0, CC2, CC3	**prob. PSP-RS**, prob. PSP-P	MAX 2

The table shows the operational values of the criteria used by Respondek et al. in 2014, the Movement Disorders Society (MDS) criteria and the MAX rules. All patients met definite postmortem diagnosis of PSP and these criteria were applyed retrospectively. Bold represents the phenotype selected after applicate the MAX rules when more than one phenotype was available.

MDS-3y, MDS criteria at 3 years of disease evolution; MDS-6y, MDS criteria at 6 years of disease evolution; MDS-last, MDS criteria at the last clinical assessment; RS, Richardson's syndrome; PI, Postural Instability; OM, Oculomotor; P, Parkinsonism; CBS, Corticobasal Syndrome; FTD, Frontotemporal Dysfunction; PNFA, Progressive Non-Fluent Aphasia; SD, Semantic Dementia; C, Cerebellar; PSP-SL, PSP with predominant speech/languaje disorder; PSP-F, PSP with predominant frontal presentation; PSP-P, predominant parkinsonism; s.o., sugestive of; poss; possible; prob, probable.

Supplementary Table 3. Disease duration comparison between PSP phenotypes.

Phenotype classification	Cox´s regression*	Hazard Ratio	95% IC Inferior	95% IC Superior	p-value
Respondek	**PSP-RS/PI** vs. rest	2.503	1.069	5.863	**0.035**
	Rest vs. **PSP-P**	5.076	0.749	34.391	0.096
MDS-3y	**PSP-RS/PI** vs. rest	2.766	0.827	9.249	0.099
	Rest vs. **PSP-P**	11.03	0.724	167.981	0.084
MDS-6y	**PSP-RS/PI** vs. rest	3.105	1.078	8.945	**0.036**
	Rest vs. **PSP-P**	2.733	0.587	12.733	0.2
MDS-last	**PSP-RS/PI** vs. rest	4.581	1.612	13.023	**0.004**
	Rest vs. **PSP-P**	4.509	1.125	18.069	**0.033**

The table shows the Cox´s regression for disease duration between PSP RS/PI vs. PSP-Cx + PSP-P (**PSP-RS/PI** vs. rest), and PSP-RS/PI + PSP-Cx vs. PSP-P (Rest vs **PSP-P**). * All of them were adjusted for age of onset and total tau burden. Respondek, criteria used by Respondek et al. in 2014; MDS-3y, MDS-PSP criteria at 3 years of disease evolution; MDS-6y, MDS-PSP criteria at 6 years; MDS-last; MDS-PSP criteria at the last clinical evaluation. PSP-RS/PI, Richardson Syndrome spectrum; PSP-Cx, PSP cortical predominant phenotypes; PSP-P, PSP with predominant parkinsonism.
